# Exploring domestication pattern in lotus: insights from dispensable genome assembly

**DOI:** 10.3389/fpls.2023.1294033

**Published:** 2023-11-16

**Authors:** Huanhuan Qi, Feng Yu, Shiyou Lü, Rebecca Njeri Damaris, Guoqing Dong, Pingfang Yang

**Affiliations:** ^1^ School of Life Science and Technology, Wuhan Polytechnic University, Wuhan, China; ^2^ State Key Laboratory of Biocatalysis and Enzyme Engineering, School of Life Sciences, Hubei University, Wuhan, China; ^3^ Department of Biological Sciences, Pwani University, Kilifi, Kenya

**Keywords:** *Nelumbo nucifera*, dispensable genome, domestication, selective signals, subgroups

## Abstract

Lotus (*Nelumbo nucifera* Gaertn.), an important aquatic plant in horticulture and ecosystems, has been cultivated for more than 7000 years and domesticated into three different subgroups: flower lotus, rhizome lotus, and seed lotus. To explore the domesticated regions of each subgroup, re-sequencing data of 371 lotus accessions collected from the public database were aligned to the genome of ‘China-Antique (CA)’. Unmapped reads were used to build the dispensable genome of each subgroup using a metagenome-like assembly strategy. More than 27 Mb of the dispensable genome in these three subgroups and the wild group was assembled, of which 11,761 genes were annotated. Some of the contigs in the dispensable genome were similar to the genomic segments of other lotus accessions other than ‘CA’. The annotated genes in each subgroup played essential roles in specific developmental processes. Dissection of selective signals in three cultivated subgroups also demonstrated that subgroup-specific metabolic pathways, such as the brassinosteroids metabolism enrichment in FL, associated with these selected genes in each subgroup and the contigs in dispensable genome nearly located in the domesticated regions of each subgroup, respectively. Our data presented a valuable resource for facilitating lotus genomic studies, complemented the helpful information to the reference genome, and shed light on the selective signals of domesticated subgroups.

## 1 Introduction

It has been well-recognized that horticultural plants, including vegetables, fruits, and ornamentals, are important for humans. Unlike crops, horticultural plants are much more diverse, and each species has unique biological features. Among the horticultural plants, lotus (*Nelumbo nucifera* Gaertn.) is widely used as a vegetable, medicinal herb, and ornamental, and might be the most crucial aquatic vegetable. Apart from its importance, lotus is also valuable in understanding the phylogeny of eudicot since it belongs to the Nelumbonaceae family, Nelumbo genus, which occupies a critical phylogenetic position. Because of its wide usage, lotus has been domesticated and cultivated for a long history and endowed with religious and cultural symbols (Guo, 2009). It also contains some unique biological features, such as seed longevity, lotus effect (self-cleaning), and thermogenesis of blossom, which are worth studying.

Different subgroups have been formed through long-term natural and artificial directional selection. They have representative economic traits, of which rhizome lotus is mainly for harvesting edible enlarged rhizomes, seed lotus mainly harvests lotus seeds, and flower lotus is mainly used for ornamental purposes. It is interesting to reveal the genomic regions related to the characteristics of different subgroups. Several studies have identified genome differentiation through population analysis and identified the selective regions that contributed to each subgroup (Li et al., 2020; Liu et al., 2020). Using high-quality reference genomes can improve the accuracy and reliability of variation detection. In addition, revealing the domestication and evolution selection mechanism from wild lotus to cultivated lotus is of great significance for targeted breeding.

Currently, ‘China Antique (CA)’ has three versions of the reference genome (CA v1, Ming et al., 2013; CA v2, Shi et al., 2020; CA v3, Qi et al., 2023). Genomes of a seed lotus cultivar ‘Taikonglian No. 3’ (TK) (Zheng et al., 2022a) as well as American lotus (Zheng et al., 2022b), are also well assembled. Each genome type has extremely unique genetic traits, and therefore, one single reference genome cannot fully reflect the complete genetic information of the species, especially in studying different subgroups within the same species. The pan-genomic study can obtain complete variation information which has been conducted in many species (Li et al., 2010b; Marroni et al., 2014; Gao et al., 2019; Bayer et al., 2020; Bian et al., 2021; Qin et al., 2021; Tang et al., 2022; Zhou et al., 2022). Considering the cost, a dispensable genome assembly strategy using low-coverage population sequencing data was successfully applied to rice (Yao et al., 2015). Identified the sequences absent from the reference genome and proved to be an essential part of the rice genome, which controls pivotal agronomic traits in rice. Although selection signals of different subgroups of lotus were identified in a re-sequencing population (Huang et al., 2018; Li et al., 2020; Liu et al., 2020), few studies have been conducted to investigate the specific genomes associated with rhizome, seed, and flower lotus.

In the present study, ‘CA’ genome (v3) (Qi et al., 2023) was applied to align re-sequencing reads of 379 lotus accessions. The unmapped reads of each subgroup were subjected to assembling its dispensable genome, and the potential mechanisms associated with domesticated lotus subgroups were further clarified. The results presented here will facilitate genomic investigations and breeding in lotus.

## 2 Results

### 2.1 Assembling of lotus dispensable genome

**Figure 1:**
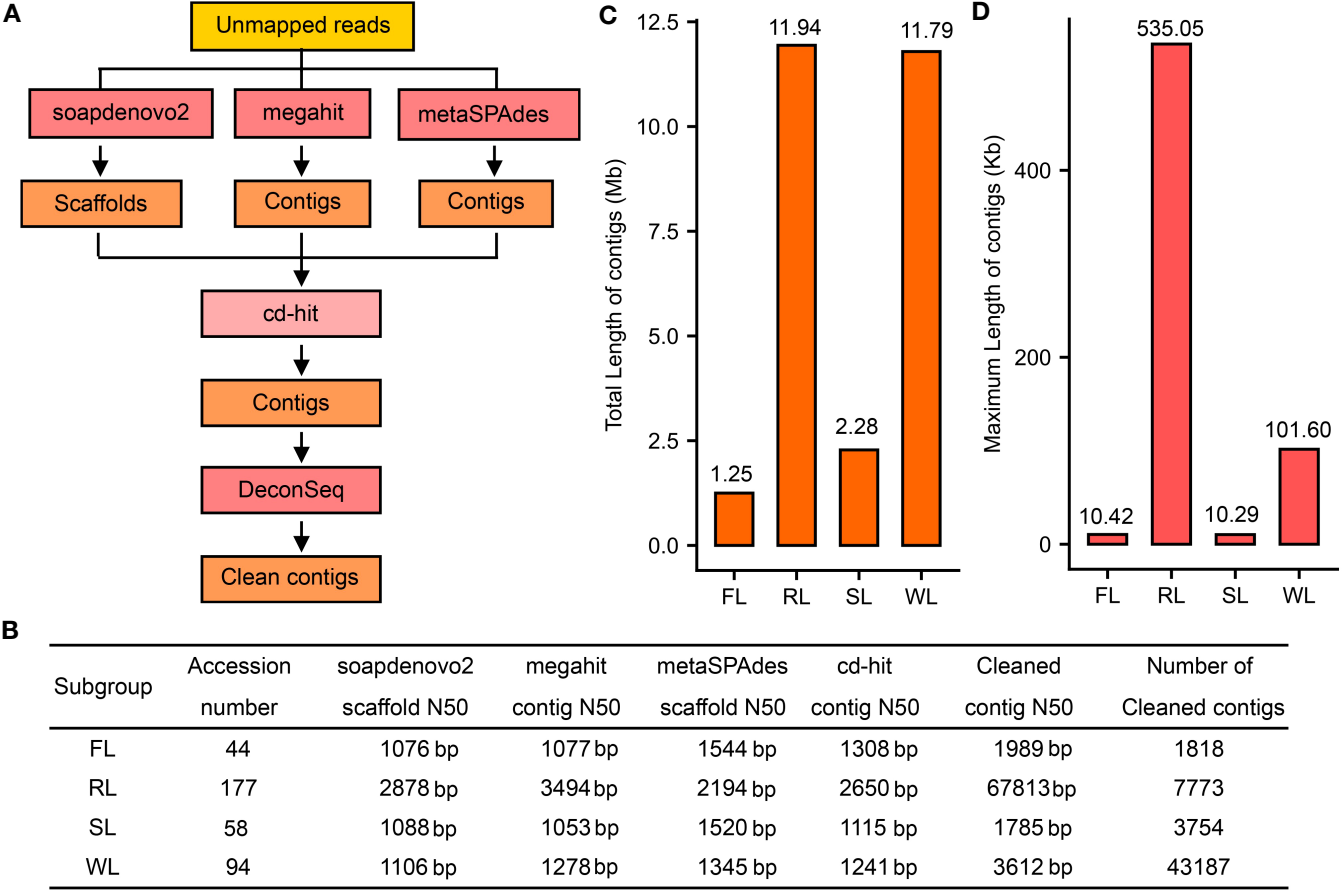
Assembly of the dispensable genome for different subgroups of lotus. **(A)**, The strategy for *de novo* assembly of the dispensable genome. **(B)**, Assembly results using different tools. **(C)**, Statistics of the total length of contigs of the dispensable genome for FL, RL, SL, and WL subgroups, respectively. **(D)**, Statistics of maximum length of contigs of the dispensable genome in FL, RL, SL, and WL, respectively. FL, flower lotus; RL, rhizome lotus; SL, seed lotus; WL, wild lotus.

To assemble the dispensable lotus genome, we collected the genome re-sequencing data for 379 lotus accessions from previous studies (Huang et al., 2018; Li et al., 2020; Liu et al., 2020). After the removal of two duplicates and 6 American lotus varieties (AL) data, a total of 371 varieties (**Table S1**) were used, which mapped to a total of 22.7 billion reads in our newly assembled genome CA v3 using BWA as the previous report (Li and Durbin, 2009a). It is worth noting that all other analyses conducted included the entire 379 lotus accessions. The unmapped reads of each accession were extracted for further analysis. The mapping rate of each accession ranged from 86.04% to 97.23% in the Asian lotus and was around 80% for the American lotus (**Table S1**). The genotype of SNPs was analyzed using GATK (McKenna et al., 2010), and a total of 15,591,976 SNPs (missing rate < 0.2 and minor allele frequency > 0.01) were finally generated. Based on these SNPs, phylogenetic tree construction and PCA analysis were conducted for these accessions to show their relationship (**Figures S1A**, **S2A**). Based on these analyses, the American lotus accessions were separated from Asian lotus. As for Asian lotus accessions, although most of them in different subgroups were well grouped, a lot of flower lotus (FL), seed lotus (SL), and wild lotus (WL) accessions were mixed (**Figures S1A**, **S2A**). The unmapped reads of well-grouped accessions that were not ambiguous in FL, RL, SL, and WL were preliminarily merged to minimize the possible error of accession grouping. The reads in each group were independently assembled using soapdenovo2 (Luo et al., 2012), megahit (Li et al., 2015), and metaSPAdes (Nurk et al., 2017) (**Figure 1A**). The resulting scaffolds from soapdenovo2 and contigs from megahit and metaSPAdes were subjected to cluster and reduced redundancy sequences. To evaluate the mapping rates. The unmapped reads of these ambiguous accessions were mapped to the primarily assembled genomes of FL, RL, SL, and WL. According to the mapping rates, twenty-one accessions were regrouped (**Table S1**). The new phylogenetic trees (**Figure S1B**) and PCA results (**Figure S2B**) based on the regrouped data were well concerted, in which 44, 177, 58, and 94 accessions were finally divided into FL, RL, SL, and WL, respectively (**Figure 1B**).

A total of 1.6 Gb, 6.2 Gb, 4.0 Gb, and 4.6 Gb unmapped sequences were finally merged for FL, RL, SL, and WL subgroups, respectively (**Table S2**) and assembled following the workflows mentioned above (**Figure 1A**). After clustering with cd-hit, contig N50 of the assembly of four subgroups ranged from 1115 bp in SL to 2650 bp in RL (**Figure 1B**). Contig N50 of the final assembly of RL was 67,813 bp, while it was 1989, 1785, and 3612 bp for the assembly of FL, SL, and WL, respectively, which were significantly improved after the removal of the contaminations using DeconSeq (**Figure 1B**). The genome of WL had apparent more contigs (43,187), whereas the contigs were less than 4000 in both FL and SL. The total length of the final contig in RL and WL (more than 11 Mb) was much longer than that in FL and SL (less than 2.3 Mb) (**Figure 1C**). The genome of RL had the longest contig (535.05 kb), while the longest contig in WL was 101.8 kb, but the longest contig in FL and SL was only approximately 10 kb (**Figure 1D**). These data indicated that the dispensable genomes of different lotus subgroups may be differential.

### 2.2 Evaluation of the assembly of dispensable genomes

**Figure 2:**
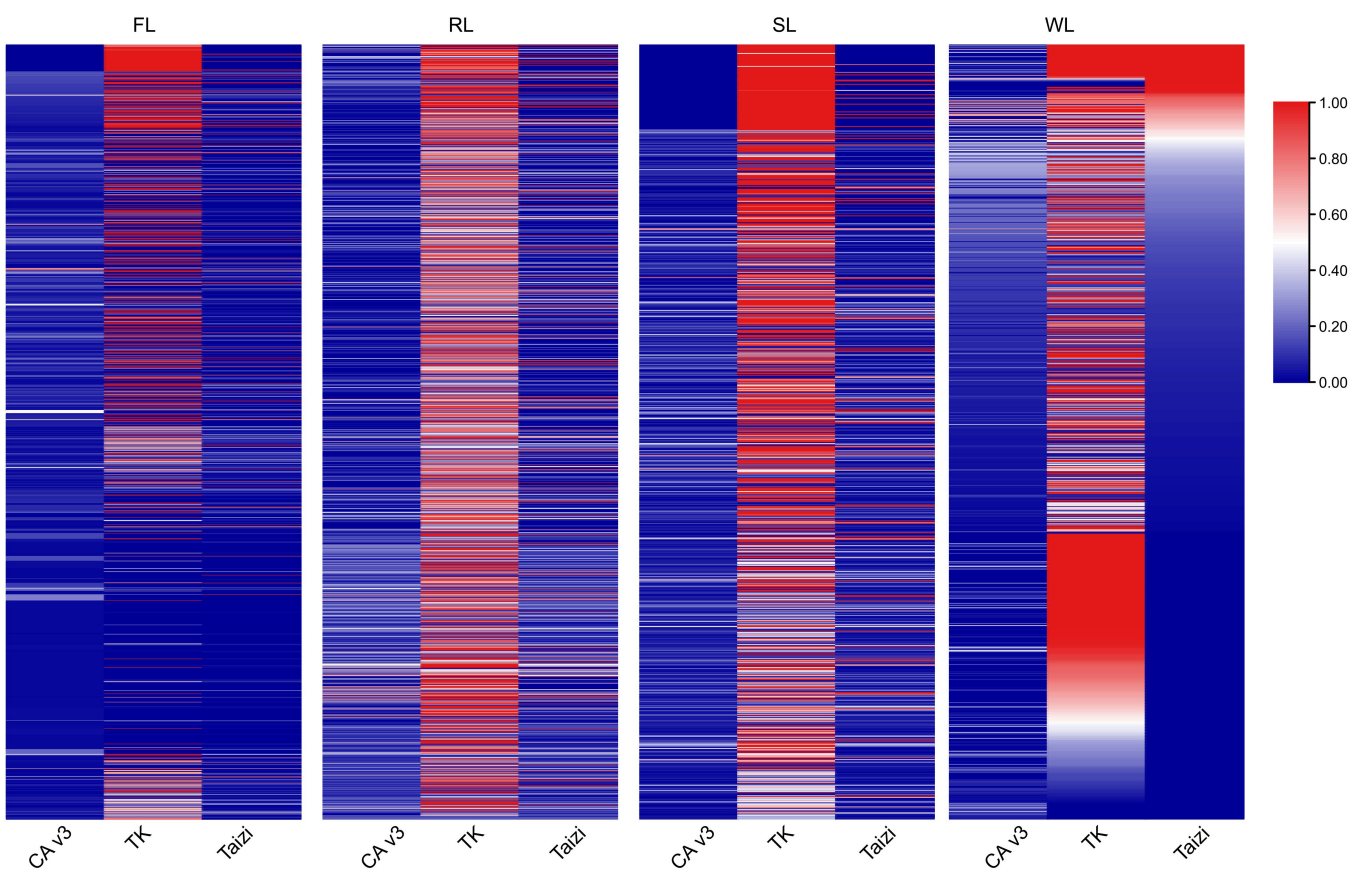
The heatmap images showing the coverage of assembled contigs aligned to the genome of CA v3, TK, and TZ, respectively. CA v3, “China Antique” v3 genome; TK, genome of ‘TaiKonglian NO.3’; TZ, genome of “TaiZi”. FL, flower lotus; RL, rhizome lotus; SL, seed lotus; WL, wild lotus. The number of the aligned contigs was 1754, 1386, 900, and 1037 in FL, RL, SL, and WL, respectively.

To evaluate the quality of the assembled dispensable genomes, all the contigs were aligned to three genome assemblies of different lotus varieties, including CA v3 (Qi et al., 2023), TK (Zheng et al., 2022a), and ‘Chinese Tai-zi’ (Wang et al., 2013), using blastn program. About 1754 of the contig in the FL dispensable genome could be matched in the three assemblies, while 1387 contig in RL and 900 contigs in SL were aligned. Still, only 2.4% of contigs in the WL dispensable genome were also aligned (**Figure 2**). Most of these aligned contigs had the highest coverage in the TK assembly. Although over 96% of the contigs in the FL dispensable genome could be aligned to the other three genomes, the contig coverage was relatively low. Moreover, more than 300 contigs of each dispensable genome with contig length 6422 bp of the longest contig (identity of most contig > 98%) could be entirely aligned to the TK genome. The coverage of more than 500 contigs was more than 0.5 (**Table S3**), implying that the dispensable genome had assembled the complete segments in the TK genome. The number of contigs with high coverage in TZ was less than in TK, but few contigs could be aligned to CA v3 with more than 0.5 (**Table S3**). These results indicated that most of the contigs were newly assembled, and the workflow used in the present study could allow the successful assembling of the dispensable genomes.

To evaluate the redundancy among the dispensable genomes of four subgroups, the blastn was applied to align one dispensable genome to the other three. The alignment hits of contig with identity ≥ 90% and match length ≥ 60% were considered similar sequences. Approximately 50% of dispensable genomes in FL and SL had alignment hits to the other three subgroups. About 14% ~ 20% of dispensable genomes in RL had alignment hits. In contrast, only 3% ~ 4% of dispensable genomes in WL had alignment hits (**Figure S3A**), indicating that most of these assembled contigs were subgroup-specific. Less than 33%, 10%, 18%, and 2% of contigs in FL, RL, SL, and WL had reciprocal coordinate overlaps with other subgroups. The contig pairs ranged from 674 to 1086 (**Figure S3B**), suggesting that most sequences of these dispensable genomes are subject to gain and loss under domestication.

### 2.3 Annotation and functional analysis of genes in dispensable genomes

**Figure 3:**
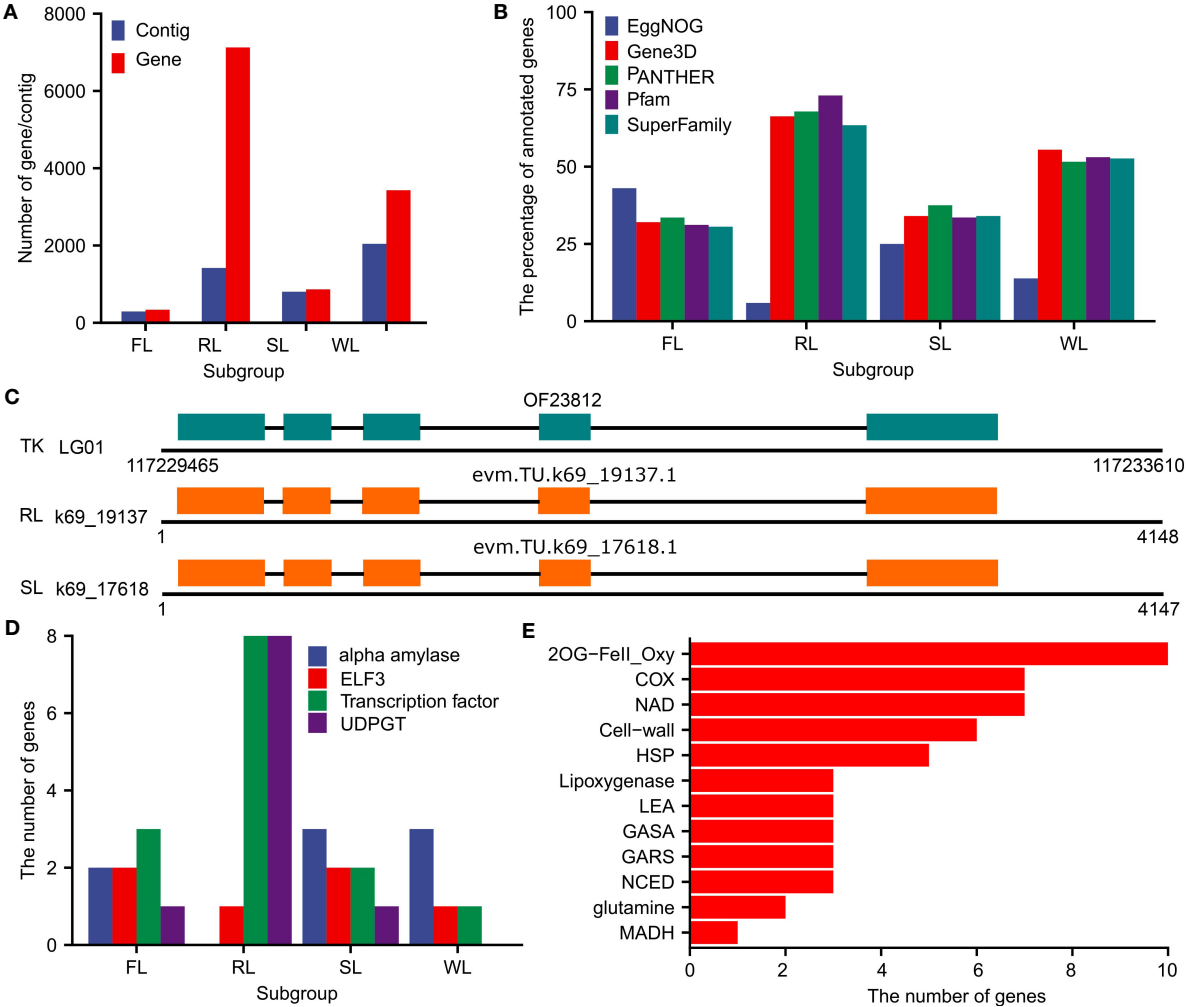
Summary of annotated genes in the dispensable genome of FL, RL, SL, and WL. **(A)**, Total number of contigs and genes in the dispensable genome of FL, RL, SL, and WL, respectively. **(B)**, The percentage of annotated genes in the database of EggNOG, Gene3D, PANTHER, Pfam, and SuperFamily in the dispensable genome of FL, RL, SL, and WL, respectively. **(C)**, Example of genes in the dispensable genome of RL and SL that was consistent with genes in TK. **(D)**, The number of genes encoding alpha-amylase, ELF3, transcription factor, UDPGT in the dispensable genome of FL, RL, SL, and WL, respectively. **(E)**, Genes associated with aquatic environment adaptation in dispensable genome of RL. FL, flower lotus; RL, rhizome lotus; SL, seed lotus; WL, wild lotus; TK, ‘TaiKonglian NO.3’; ELF, protein early flowering; UDPGT, UDP-glycosyltransferase.

The annotation pipeline combing Augustus (v3.4.0, Stanke et al., 2006), geneid (v1.4, Parra et al., 2000), genome Threader (v1.7.3, Gremme et al., 2005), PASA and EvidenceModeler (v1.1.1) was applied to predict protein-coding genes in the dispensable genomes. The numbers of predicted genes were 337 (in 293 contigs), 7126 (in 1419 contigs), 864 (in 804 contigs), and 3430 (in 2043 contigs) for the dispensable genomes of FL, RL, SL, and WL, respectively (**Figure 3A**; **Table S4**). These predicted genes were further annotated by searching against the databases of EggNOG, Gene3D, PANTHER, Pfam, and SuperFamily. The results showed that approximately 30%, 75%, 40%, and 50% of the predicted genes could be annotated in at least one database of Gene3D, PANTHER, Pfam, and SuperFamily for FL, RL, SL, and WL, respectively (**Figure 3B**). The annotated results from EggNOG differed from the other four databases, of which 43%, 5.9%, 25%, and 13.8% of genes were annotated in FL, RL, SL, and WL, respectively (**Figure 3B**). To check the reliability of these predicted genes, we selected the contigs that could be fully aligned to the genome assembly of TK for validation. We found that approximately 40% of the predicted genes were also annotated as protein-coding genes in TK (**Figure S4**), with many of them showing the same gene structures (**Figure 3C**). We also aligned the protein sequences in dispensable genomes against Arabidopsis data using blastp program, which indicated that 29.4%, 36.2%, 31.3%, and 35.2% of the proteins in dispensable genomes of FL, RL, SL, and WL had their homologs in Arabidopsis, respectively (**Table S4**). Moreover, some predicted genes were annotated as biological functional important ones, including alpha-amylase, ELF3, transcription factor, and UGT (**Figure 3D**). Specifically, a lot of gene families involved in adapting to abiotic stress (aquatic environment) were identified in the dispensable genome of RL, which included ten members of iron ascorbate-dependent oxidoreductase family (2OG-FeII_Oxy), seven members of cytochrome c oxidase family (COX), seven members of NADH dehydrogenase (NAD), six members related to cell-wall, and anaerobic metabolism related members of glutamine and malate dehydrogenase (MADH) (**Figure 3E**). These data collectively demonstrated that the predicted genes in dispensable genomes were precise and had an essential function.

### 2.4 Identification of the regions and genes under selection

**Figure 4:**
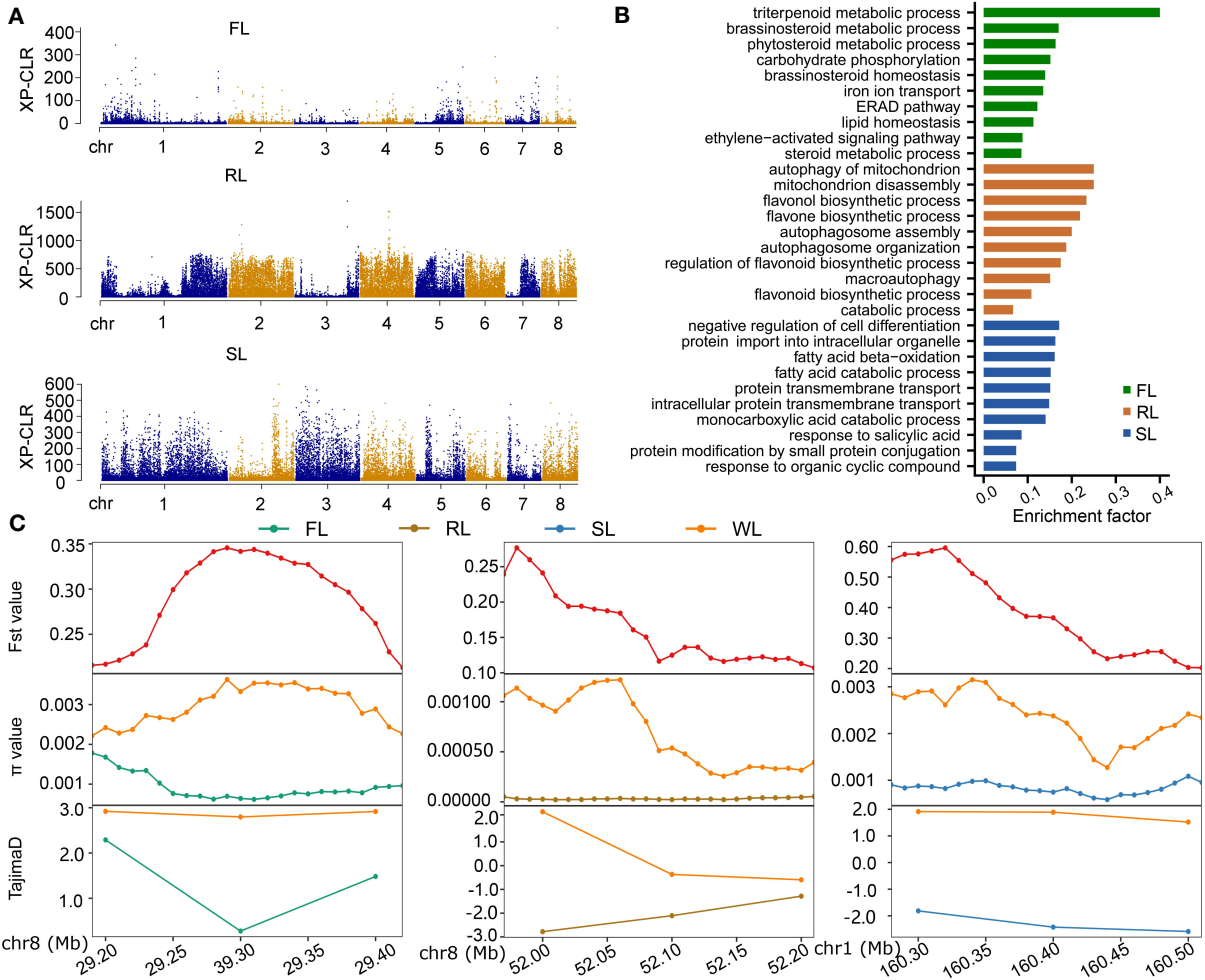
Determination of the genetic regions subjected to domestication selection in different subgroups of lotus. **(A)**, Manhattan plots showing XP-CLR scores spanning chromosomes. **(B)**, KEGG enrichment of genes within selected regions of FL, RL, and SL. **(C)**, The value of Fst, π, and Tajima’s D in three randomly selected regions identified from XP-CLR. FL, flower lotus; RL, rhizome lotus; SL, seed lotus; WL, wild lotus; XP-CLR, cross-population composite likelihood ratio test; Fst, F-statistics.

To identify selective sweep regions, the cross-population composite likelihood ratio test (XP-CLR) was used to compare the WL subgroup with the other three subgroups, respectively. A total of 207, 274, and 269 putative windows in FL, RL, and SL were detected using the threshold of 1% cutoff of XP-CLR scores, respectively (**Figure 4A**). Of these windows, 923, 1302, and 1332 genes in FL, RL, and SL were identified, respectively (**Table S5**). The most significant scores in FL were located within two intervals: chr1, 25,340,001-25,440,000 and chr8, 29,260,001-29,360,000, encoding genes *NNU01g00704* and *NNU08g00595*, respectively. The *NNU08g00595* has GABA-A receptor activity, which is involved in the gamma-aminobutyric acid signaling pathway. Windows with the most significant scores in RL were in chr2, chr3, and chr4, while those in SL were mainly located in chr2 and chr3. Some candidate genes located in these selected regions play essential roles in seed and rhizome development, such as *NNU03g03304* and *NNU04g01294* of RL being involved in starch and sucrose metabolism and oxidation-reduction, respectively, and *NNU03g01108*, *NNU03g011011*, *NNU03g01111*, and *NNU03g02278* of SL being involved in primary metabolism processes. Most of the selected genes in FL, RL, and WL were subgroup-specific, and only four genes (*NNU06g01700*, *NNU06g01701*, *NNU06g01702*, and *NNU06g01703*) were commonly chosen for three subgroups, while about 20~30 genes were commonly selected by two subgroups (**Figure S5**). The selected genes in each subgroup were further subjected to KEGG enrichment analysis, based on which the brassinosteroid and ethylene metabolism and signaling were enriched in FL, and the autophagy- and flavone-related processes were enriched in RL. The primary metabolic processes, such as the fatty acid catabolic process and salicylic acid-responsive process were enriched in SL (**Figure 4B**). We randomly selected three domesticated regions determined by XP-CLR to analyze the nucleotide diversity through π value, Tajima’s D, and Fst value. The results demonstrated apparent differentiation between domesticated subgroups (FL, RL, and SL) and wild subgroups (**Figure 4C**), suggesting that the approach of XP-CLR was suitable for identifying selected signals in the lotus genome.

### 2.5 The positions of the assembled sequence located nearly with domestication regions

**Figure 5:**
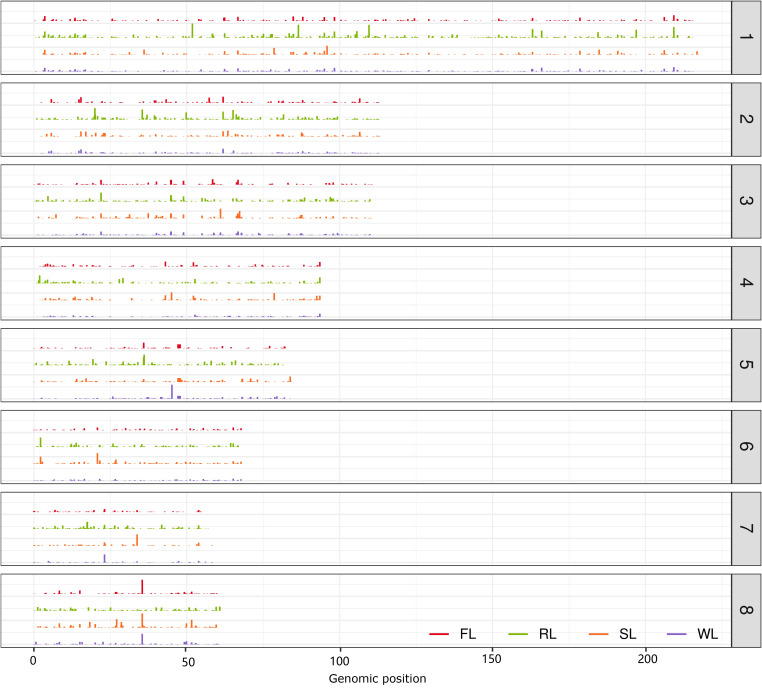
The distribution of aligned contig of the dispensable genomes on the eight chromosomes. The eight chromosomes were arranged from top to bottom. The height of the bars indicates the length of the aligned contig of the dispensable genome in FL, RL, and SL, respectively.

Since the contigs of the dispensable genome assembled from some hanging read pairs could be partially aligned to the CA v3 genome, many contigs contained a proportion of sequences that could be found in the reference genome. To determine the position of the contigs on the CA v3 genome, each contig was aligned to CA v3 using the blastn program, and the hits with the top two highest scores were retained (**Figure 5**). A total of 598, 930, 663, and 722 contigs in FL, RL, SL, and WL were assigned to 1366, 2402, 1687, and 1876 genomic regions, respectively. The distribution of the dispensable locating positions along the chromosomes was not well coordinated with the length of the chromosome, in which the most prominent amount of positions located in chromosome 3 and the least amount of positions located in chromosome 7 (**Figure S6**), suggesting that distribution of these contigs were specific. To validate whether the positions of these contigs in CA v3 were associated with selected regions, we compared the genomic positions of FL, RL, and SL contig with their corresponding selected regions, respectively. The results demonstrated about 5% genomic positions of dispensable contig located in selected regions, and approximately 15% genomic positions of dispensable contig found in 200 kb flanking selected regions (**Figure S7**). The proportions increased with the broadening flanking regions. Almost all these genomic positions of dispensable contigs were located in 2 Mb flanking selected regions, indicating that the contig of each dispensable genome is located nearly with domesticated regions of each subgroup.

## 3 Discussion

Lotus has been cultivated in Asia for over 7000 years and domesticated into three different subgroups: FL, RL, and SL, based on the specific characteristics of flower, rhizome, and seed, respectively (Ming et al., 2013). Although many studies have been conducted to illuminate causal factors of different agronomic traits (Huang et al., 2018; Li et al., 2020; Liu et al., 2020; Qi et al., 2021), few works were done to clarify the dispensable genome in each subgroup. A single reference genome was inefficient in identifying whole genetic variations within one species, limiting the genetic improvement in crops (Tao et al., 2019). Recent advancements in sequencing technologies and bioinformatic tools facilitated the assembly of multiple genomes with high quality in a species, although it is expensive (Golicz et al., 2016). Alternatively, a metagenome-like assembly strategy using low-coverage population sequencing data has been developed and proved feasible in constructing dispensable genomes in rice (Yao et al., 2015). In benefit from re-sequencing accessions of lotus (Huang et al., 2018; Li et al., 2020; Liu et al., 2020), we attempted to assemble the dispensable genomes of FL, RL, SL, and WL using dispensable sequences that could not be aligned to genome CA v3 based on the metagenome-like assembly strategy (**Figure 1**). Fortunately, over 27 Mb of the dispensable genome were assembled, and the longest contig was more than 500 kb. Specifically, some parts of these contigs had high coverage with the genome of TK and TZ (**Figure 2**; **Table S3**). Further annotation of protein-coding genes also demonstrated that these dispensable genomes were functionally crucial because many genes were predicted in each dispensable genome (**Figure 3**). Although there is no direct evidence to verify the vital function of the genes in dispensable genomes, some clues presented in our results manifested their functional importance in each subgroup, which included 1) a lot of genes in the dispensable genome could be annotated in 5 databases; 2) some genes predicted in a contig of the dispensable genome were also annotated as protein-coding genes in TK genome; 3) about 33% in average proteins in the dispensable genome were similar to Arabidopsis proteins; 4) some genes such as stress-responsive genes in the dispensable genome of RL were assembled. These results indicated that our strategy could successfully assemble dispensable contigs contained in other genomes, which would be helpful for subgroup-specific functional investigation. However, the length of the dispensable genome in RL and WL was more prolonged than in FL and SL. The difference in the accession numbers in each subgroup may be the main reason causing variations in the length of dispensable genomes since the accession number of each subgroup seems positively correlated with total length. However, the size of the dispensable genome in RL was similar to WL. The accession numbers in WL were approximately half of that in RL. These results suggested that the accession number in each subgroup was not the only determinative factor. The number of unmapped reads might correlate with the final assembly. Similar amounts of unmapped reads in SL and RL subgroups (**Table S2**) yielded a 5-fold difference in total length, indicating that the amount of sequence data also did not directly correlate with the full size of dispensable genomes. Moreover, the number of contigs in the dispensable genome of WL was higher than those in other subgroups. In contrast, the number of annotated genes in WL was less than half of those in RL, implying that the sequence variations in WL were higher than in RL. This is reasonable because the lotus propagated through asexual reproduction. In most cases, the variations in WL were retained. Using the above-mentioned strategy in lotus in the future, the accession number of each subgroup, the data amount, and the specific characters of different subgroups need to be taken into account, promoting the assembly of dispensable genomes with high quality. However, when comparing two high-quality genome, CA v3 and TK, approximately 33 Mb sequence in CA v3 were not aligned to TK genome and about 24 Mb sequence in TK were also not aligned to CA v3 genome (**Table S6**), These findings suggested potential missing assemblies in our pipelines and underscored the need for more well-assembled genomes for comprehensive pan-genome analysis.

The cultivated lotus was domesticated from wild accessions through cross-breeding and artificial selection. This may have resulted in reduced nucleotide diversity and maintenance of the signals associated with specific traits, making identifying candidate regions correlated with selected signals possible. Based on the combination of Fst and π values, 24, 77, and 2176 genes were selected in a subgroup of FL, RL, and SL compared with WL in the population of 69 accessions (Li et al., 2020). Some of these selected genes contributed to the essential domestication traits, including the selected genes of SL, primarily affecting seed weight and size, and the genes of RL, mainly improving the size of the rhizome. However, less domesticated genes were identified in the subgroup of FL and RL. It may be caused by the mini size of the population and the low quality of the reference genome (CA v1), which directly affected the nucleotides’ accuracy and frequency. Moreover, single locus Fst values are highly variable, and Fst measurements in sliding windows do not take advantage of the detailed pattern of allele frequency in a selected region (Weir et al., 2005). XP-CLR was another statistical method for detecting selective sweeps based on multi-loci frequency differentiation between two populations (Chen et al., 2010), which was widely applied in the identification of selected regions of cultivated populations (Hufford et al., 2012; Wang et al., 2020; Chen et al., 2022). Using 371 lotus accessions collected for assembling dispensable genomes, the XP-CLR method was applied to identify selective signals in the FL, RL, and SL subgroups, which were well coordinated with Fst, π, and Tajima’s D approaches (**Figure 4**). The number of selected genes in each subgroup differed from previous results (Li et al., 2020). Few genes were simultaneously selected in three subgroups, and few genes were selected in any two subgroups, indicating that the selection of each subgroup was independent. Functional analysis of these genes demonstrated that genes in each subgroup presented specific functions, including BRs metabolism in FL, flavonoid and autophagy process in RL, and primary metabolism in SL, suggesting that the selection of these genes in each subgroup is associated with phenotypic traits. BRs are polyhydroxylated steroid phytohormones, and BR biosynthetic mutants *det2* in Arabidopsis exhibited delayed flowering time (Li et al., 2010a), while BR biosynthesis gene bsl1 in *Setaria viridis* is required for organ fate decisions during inflorescence development and affects morphological variation inflorescence architecture (Yang et al., 2018), indicating the essential roles of BRs in floral formation and development. The BRs metabolic process was enriched in selected genes of the FL subgroup, implying that BRs are also an important factor affecting the floral development of lotus. Interestingly, the contig of the dispensable genome of each subgroup is nearly located with domesticated genomic regions (**Figure 5**), which provides a new way of discovering the nonexistent genes in the reference genome, aiding in investigating genomic function.

## 4 Conclusion

As a basal eudicot species, lotus (*Nelumbo nucifera* Gaertn) is one of the relict plants retaining the original morphology of its ancestors. It has an evolutionary history of approximately 135 million years and is essential in studying plant evolution and phylogeny. In addition, it is also an important aquatic horticultural plant with more than 4500 cultivars or accessions, which could be differentiated into three types: FL, RL, and SL. Based on CA v3, about 1.25, 11.94, 2.28, and 11.79 Mb of the dispensable genome in FL, RL, SL, and WL subgroup was successfully assembled using public re-sequencing data of 371 accessions. Large quantities of protein-coding genes in dispensable genomes were annotated, most of which were important in subgroup-specific development. These results suggested the feasibility of building the dispensable genome of a species using population sequencing data. Moreover, lots of selected signals in FL, RL, and SL were detected, and candidate genes associated with these signals in each subgroup enriched in specific metabolic pathways such as BRs metabolism in FL. The results yielded in the present study will help understand the lotus genomic characters and subgroup-specific genomes, promoting the pick of candidate genes associated with variations of agronomic traits and expounding the differential mechanism of different cultivars.

## 5 Materials and methods

### 5.1 Variant calling, phylogenetic, and PCA analyses

The raw data of 379 lotus accessions were collected from the public database with accessions of CNP0001227 in CNGB (Liu et al., 2020) and SRP095218 in NCBI (Li et al., 2020) and from our previously sequenced data (Huang et al., 2018). The fastp (0.20.1) (Chen et al., 2018) was used to filter low-quality reads, and the clean data were mapped to the newly assembled genome of lotus with the BWA (0.7.17) software (Li and Durbin, 2009a). SAMtools (Li et al., 2009b) was employed to convert the sam format files to bam format files and further sorted them. The unmapped reads were also extracted through SAMtools. Picard (2.25.4) software was used for removing PCR duplicate reads. The Flagstats program in SAMtools was used for computing the mapping rate and coverage rate. Variation calling was performed with the Genome Analysis Toolkit (GATK, version 4.2.2.0) (McKenna et al., 2010), and HaplotypeCaller was employed to identify SNPs and indels in each accession, then GenotypeGVCFs were used for population variation detection. Low-quality variants were filtered with varianFilteration. The Plink software (Purcell et al., 2007) was applied to identify non-missing SNPs with MAF < 0.01 and missing rate > 0.5 and was used to conduct PCA analysis. Fasttree was used to construct a phylogenetic tree, and iTOL was used to visualize the trees.

### 5.2 Identification of the regions and genes under selection

The cross-population composite likelihood ratio (XP-CLR) method was employed to identify selected regions, and the parameters were set as follows: –ld, 0.95; –maxsnps, 200; –size, 100000; –step 20000. The top 1% XP-CLR scores were set as the candidate selection regions. The VCFtools (Danecek et al., 2011) was used to calculate the nucleotide diversity (π), divergent variation (Fst), and Tajima’s D among different subpopulations. The genes located in the selected region were regarded as having undergone selection. Annotation and enrichment analysis of the KEGG pathway was performed for candidate genes.

### 5.3 Dispensable genome assembly and annotation

Based on phylogenetic analysis and phenotypes, the 379 accessions were categorized into four subgroups: FL, RL, SL, and WL. All the unmapped reads of these accessions were collected and merged within each subgroup. Then the merged reads were aligned to the new assembly genome. Three software included SOAPdenovo2 (v2.04, Luo et al., 2012), megahit (v1.2.9, Li et al., 2015), and metaSPAdes (v3.15.3, Nurk et al., 2017) were applied to assemble these unmapped reads to generate contig. The cd-hit (Fu et al., 2012) nmdthg was used to combine the results from three pipelines, and then DeconSeq (v0.4.3) (Schmieder and Edwards, 2011) was used to remove contamination. The final contigs were aligned to CA v3, TK, and TZ, and the coverage of each contig to these three genomes was compared. Moreover, the potential insertion sites of these contigs in CA v3 were shown through shinyChromosome (Yu et al., 2019). To annotate these contigs (> 300 bp), Augustus (v3.4.0, Stanke et al., 2006) and geneid (v1.4, Parra et al., 2000) were used to predict protein-coding genes *de novo*, and genome Threader (v1.7.3, Gremme et al., 2005) was used to predict homology proteins while PASA was used to integrate transcriptome evidence as described above. These predicted results from Augustus, geneid, Genome Threader, and PASA were combined by EvidenceModeler (v1.1.1). Finally, EggNOG, Gene3D, PANTHER, Pfam, and SuperFamily were subjected to identify their potential functional domains.

## Data availability statement

The original contributions presented in the study are included in the article/**Supplementary Files**. Further inquiries can be directed to the corresponding authors.

## Author contributions

HQ: Writing – original draft, Writing – review and editing, Visualization. FY: Methodology, Writing – review and editing, Visualization. SL: Methodology, Writing – review and editing. RD: Writing – review and editing. GD: Conceptualization, Supervision, Writing – review and editing. PY: Methodology, Supervision, Writing – review and editing.

## Supplementary material

Supplementary Figure 1 Phylogenetic relationship of different lotus cultivars based on SNP genotypes. **(A)**, The lotus accessions were grouped according to the previously reported work. **(B)**, The lotus accessions were re-grouped based on the description in this manuscript.

Supplementary Figure 2 Principal component analysis (PCA) of lotus accessions based on SNP genotypes. **(A)**, The subgroups of lotus accessions were derived from previously reported work. **(B)**, The subgroups of lotus accessions were corrected based on genotypes.

Supplementary Figure 3 The comparison of the overlapped contig among dispensable genomes in FL, RL, SL, and WL. **(A)**, The percentage of overlapped contig among dispensable genomes in FL, RL, SL, and WL. **(B)**, The number of contig that were reciprocal coordinate overlap with other subgroups.FL, flower lotus; RL, rhizome lotus; SL, seed lotus; WL, wild lotus.

Supplementary Figure 4 The number of genes in contigs of dispensable genomes and TK, in which contigs were completely aligned with the TK genome. TK, ‘TaiKonglian NO.3’.

Supplementary Figure 5 Veen diagram showing the gene numbers in selected regions in FL, RL, and SL. FL, flower lotus; RL, rhizome lotus; SL, seed lotus.

Supplementary Figure 6 The number of genomic positions of contigs in dispensable genomes in each chromosome.

Supplementary Figure 7 The ratio of genomic positions of contigs in dispensable genomes located in or near domesticated regions.
