# Supplementary figures and images for: Exploring domestication pattern in lotus: insights from dispensable genome assembly
Source: Front Plant Sci. 2023 Nov 16;14:1294033. doi: 10.3389/fpls.2023.1294033 (PMC10687544; doi:10.3389/fpls.2023.1294033)

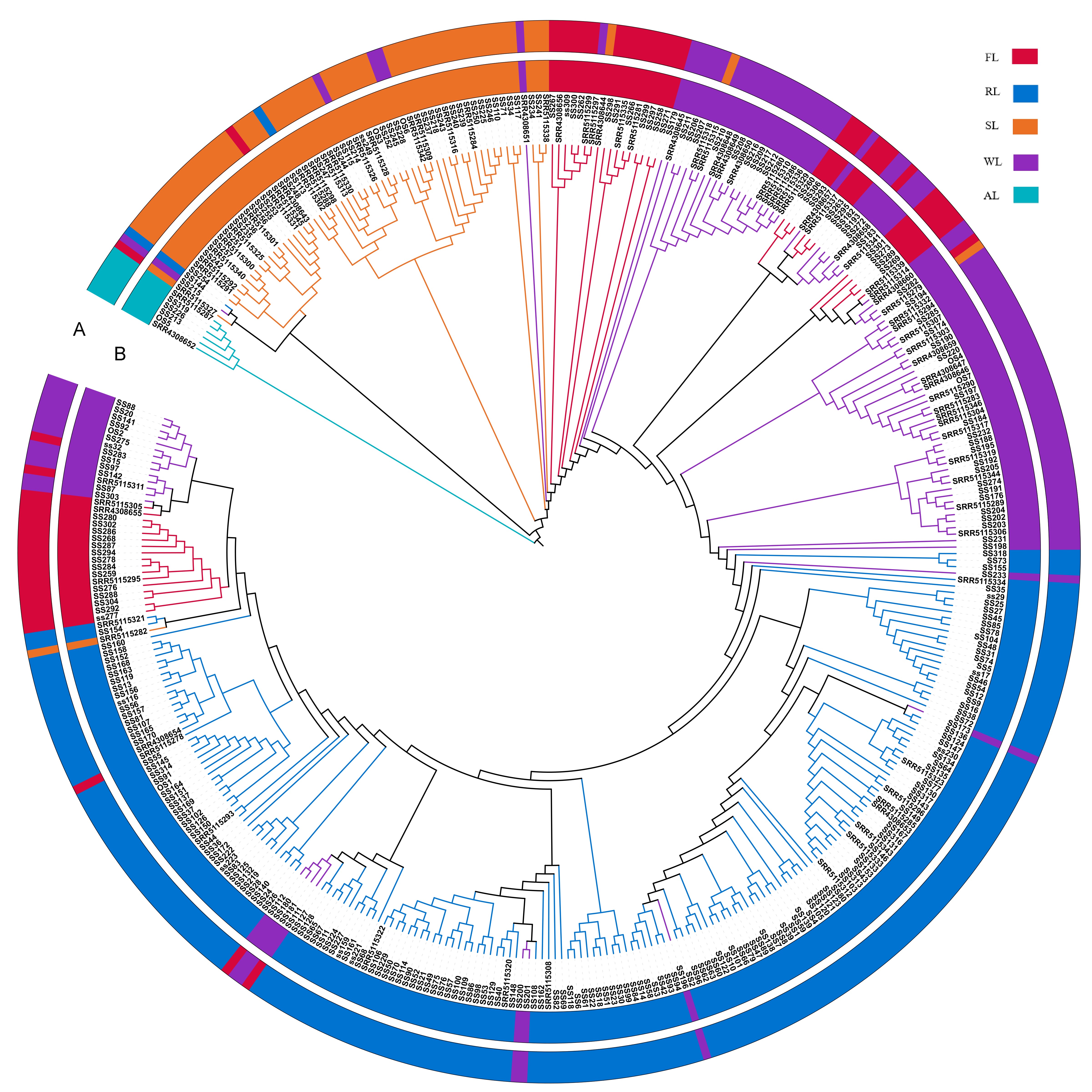

Supplement: Supplementary Figure 1 — Phylogenetic relationship of different lotus cultivars based on SNP genotypes. (A), The lotus accessions were grouped according to the previously reported work. (B), The lotus accessions were re-grouped based on the description in this manuscript. [file Image_1.jpeg]

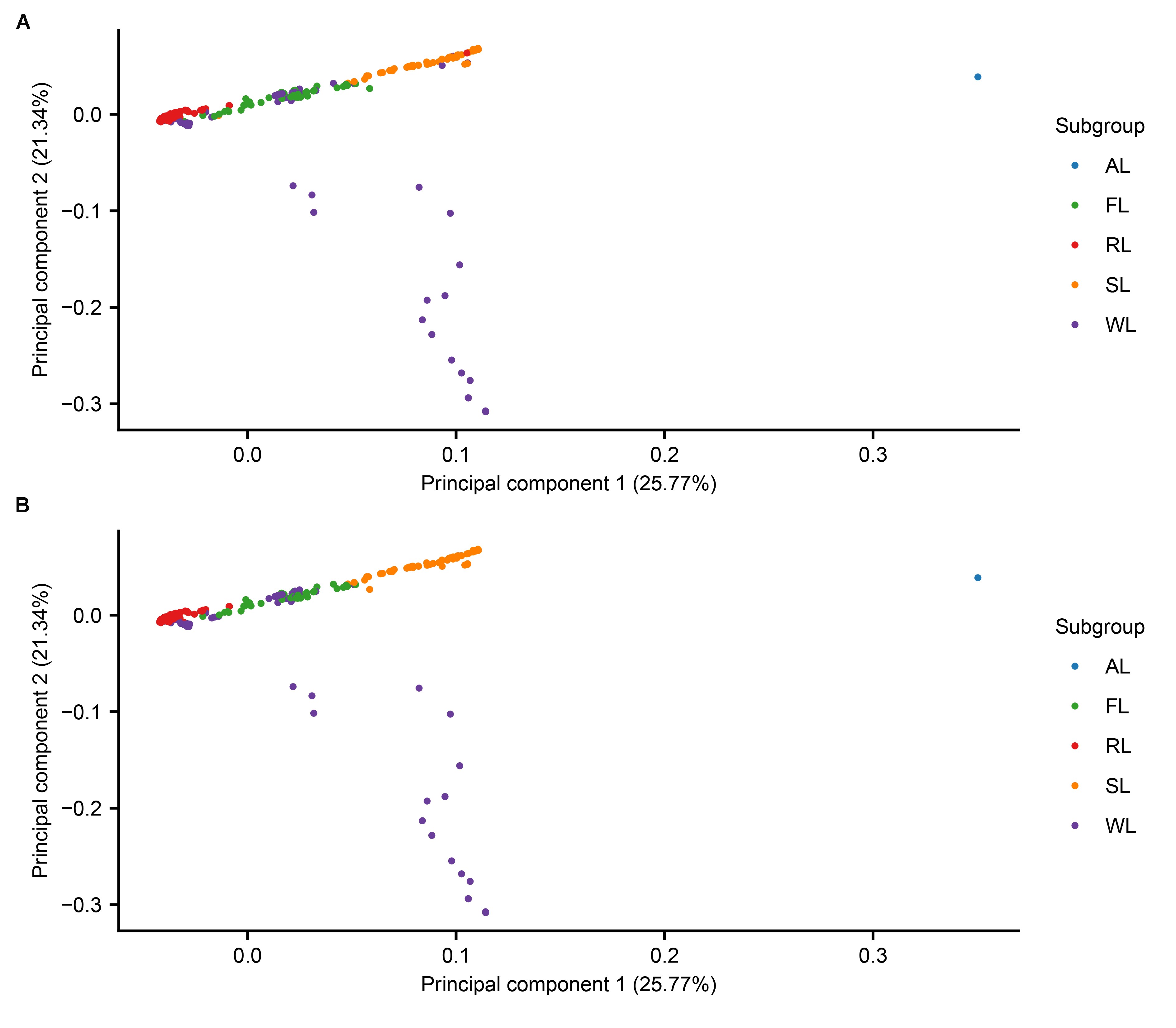

Supplement: Supplementary Figure 2 — Principal component analysis (PCA) of lotus accessions based on SNP genotypes. (A), The subgroups of lotus accessions were derived from previously reported work. (B), The subgroups of lotus accessions were corrected based on genotypes. [file Image_2.jpeg]

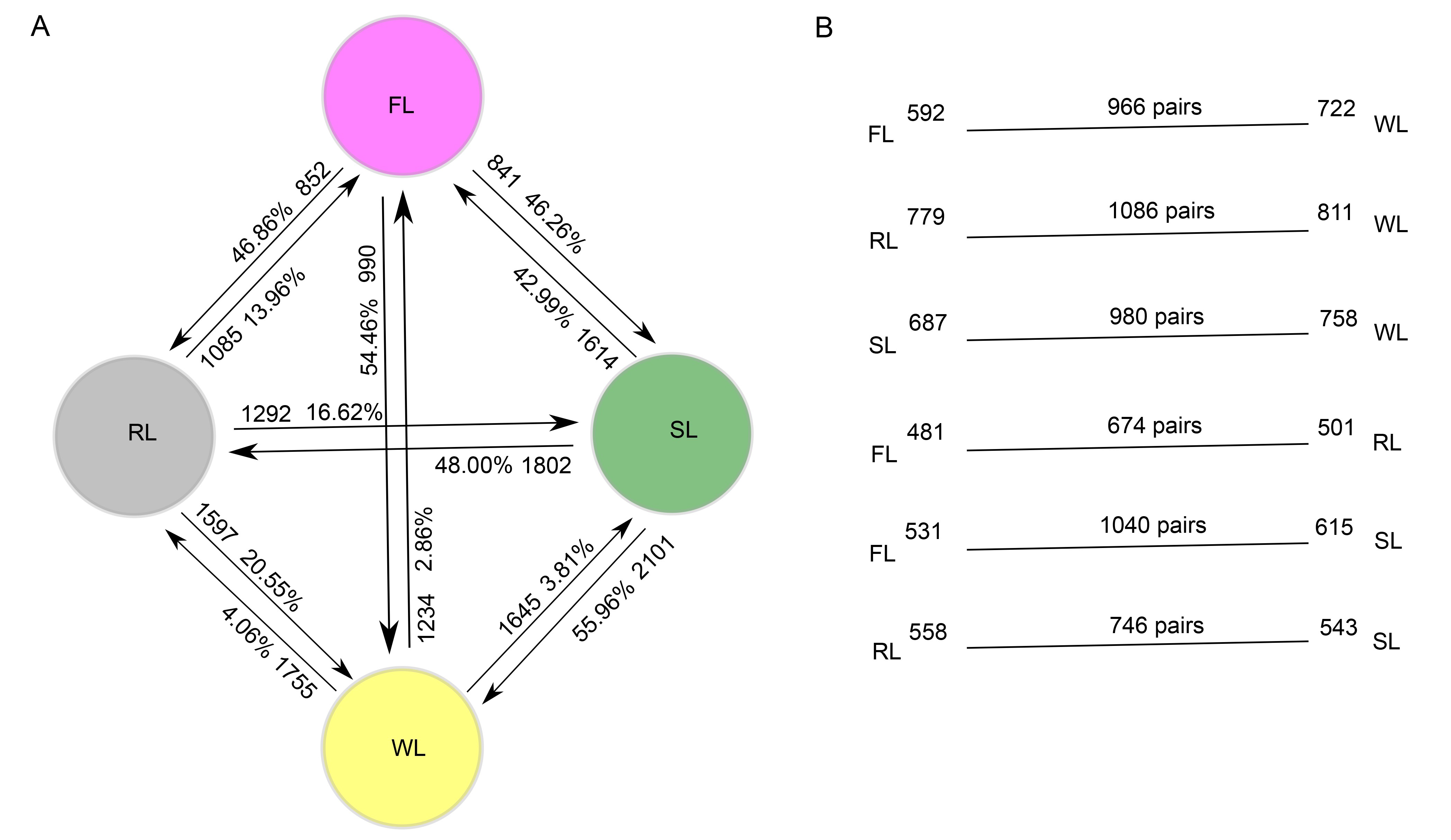

Supplement: Supplementary Figure 3 — The comparison of the overlapped contig among dispensable genomes in FL, RL, SL, and WL. (A), The percentage of overlapped contig among dispensable genomes in FL, RL, SL, and WL. (B), The number of contig that were reciprocal coordinate overlap with other subgroups.FL, flower lotus; RL, rhizome lotus; SL, seed lotus; WL, wild lotus. [file Image_3.jpeg]

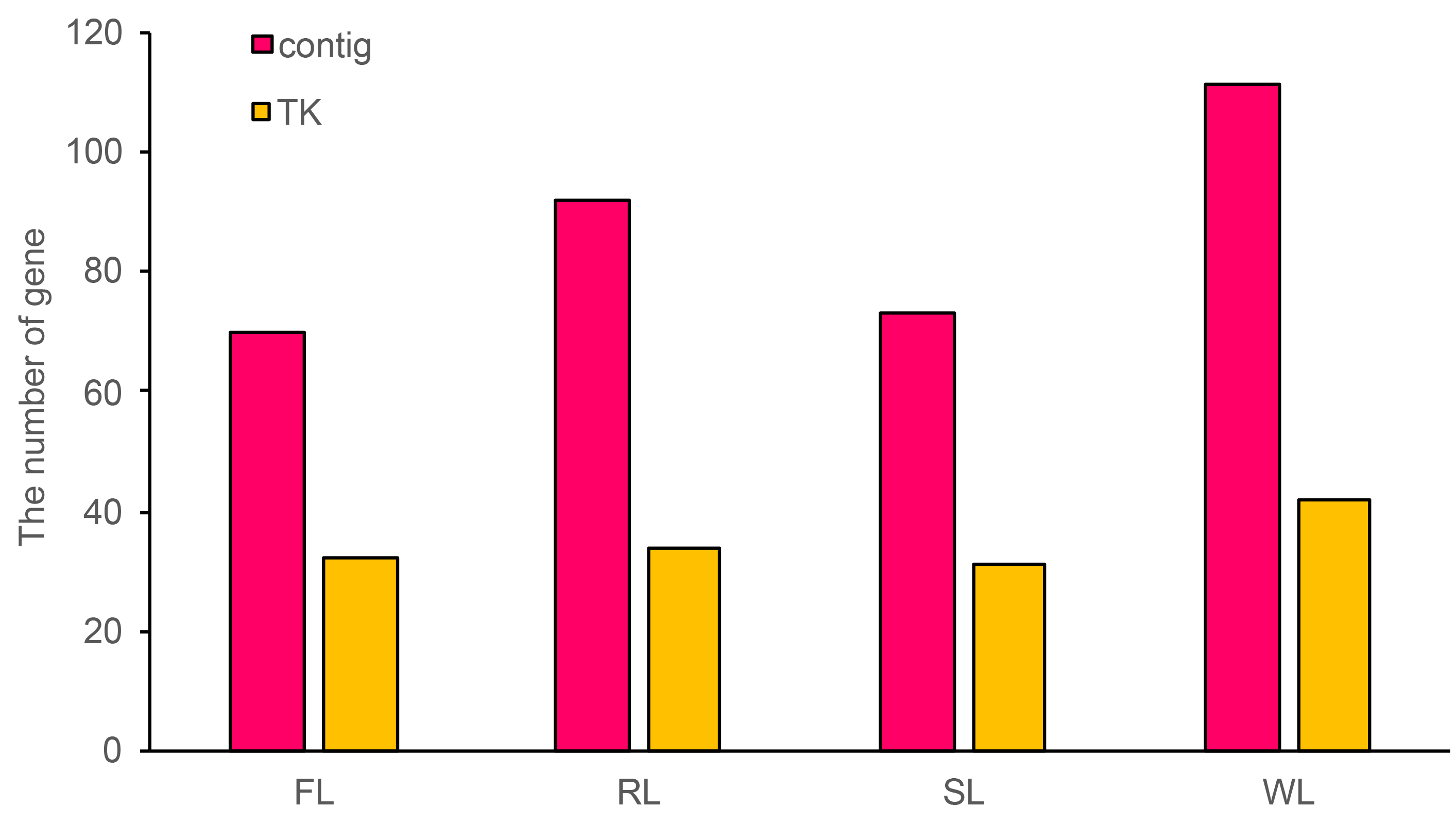

Supplement: Supplementary Figure 4 — The number of genes in contigs of dispensable genomes and TK, in which contigs were completely aligned with the TK genome. TK, ‘TaiKonglian NO.3’. [file Image_4.tif]

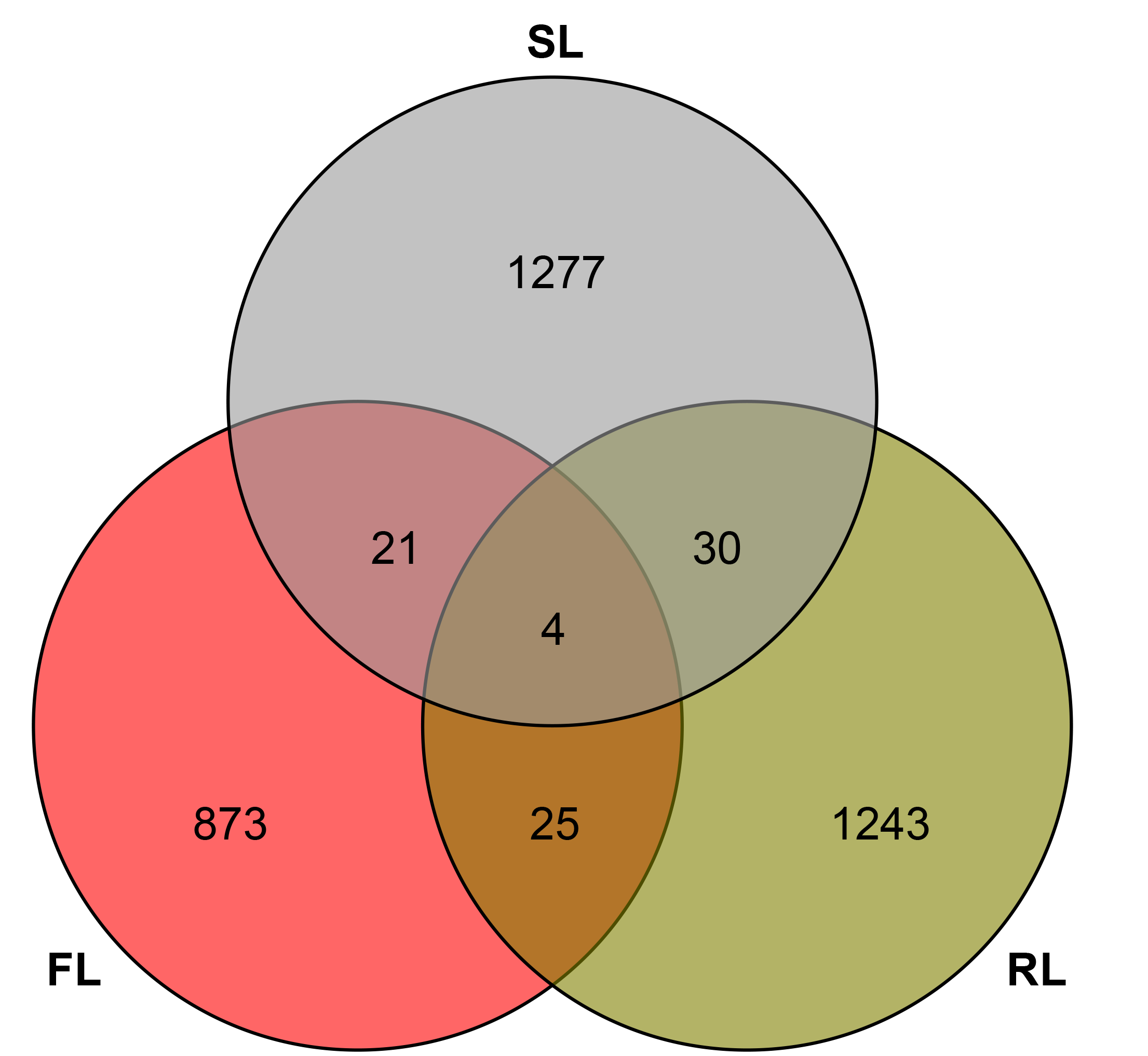

Supplement: Supplementary Figure 5 — Veen diagram showing the gene numbers in selected regions in FL, RL, and SL. FL, flower lotus; RL, rhizome lotus; SL, seed lotus. [file Image_5.tif]

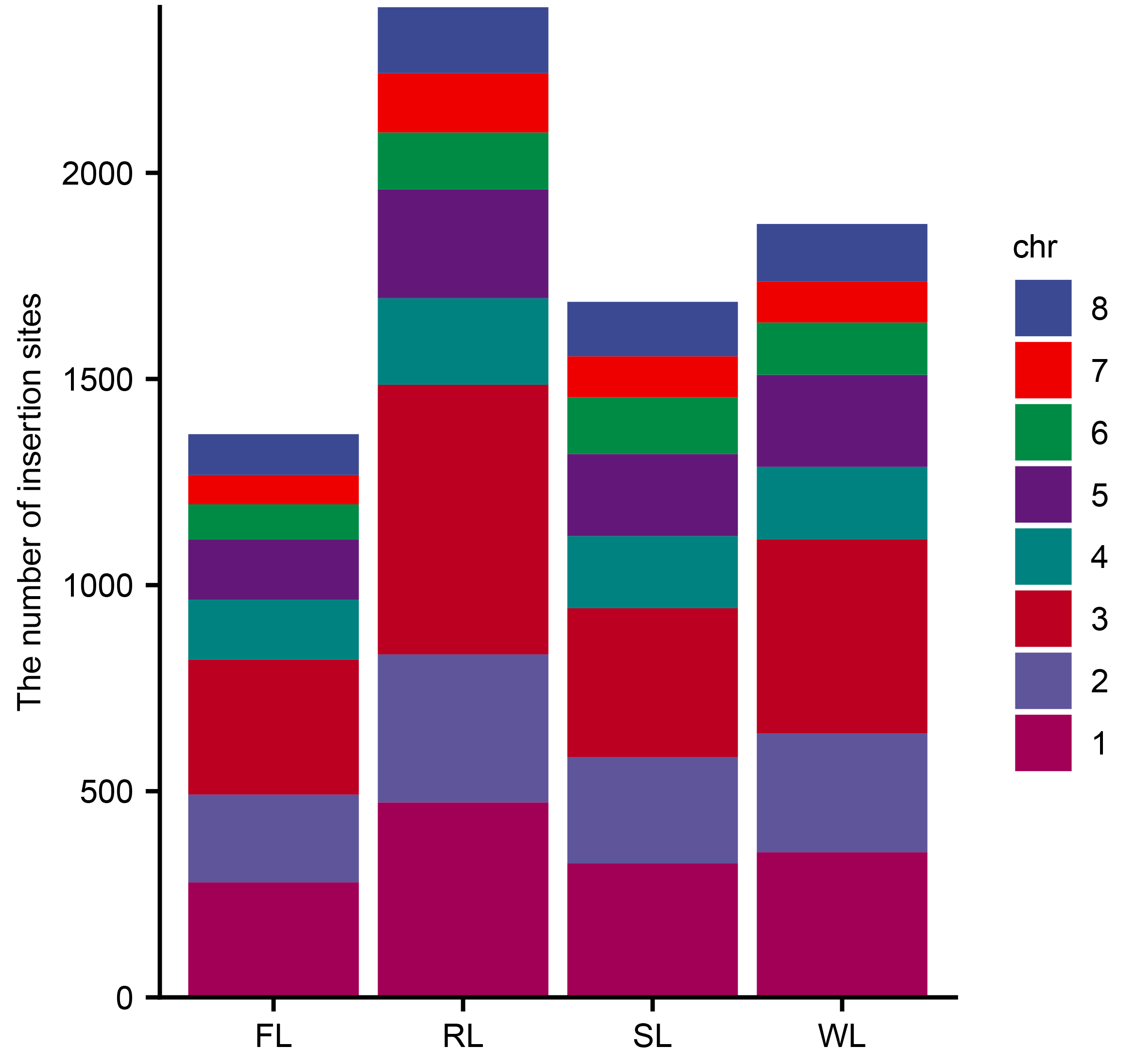

Supplement: Supplementary Figure 6 — The number of genomic positions of contigs in dispensable genomes in each chromosome. [file Image_6.tif]

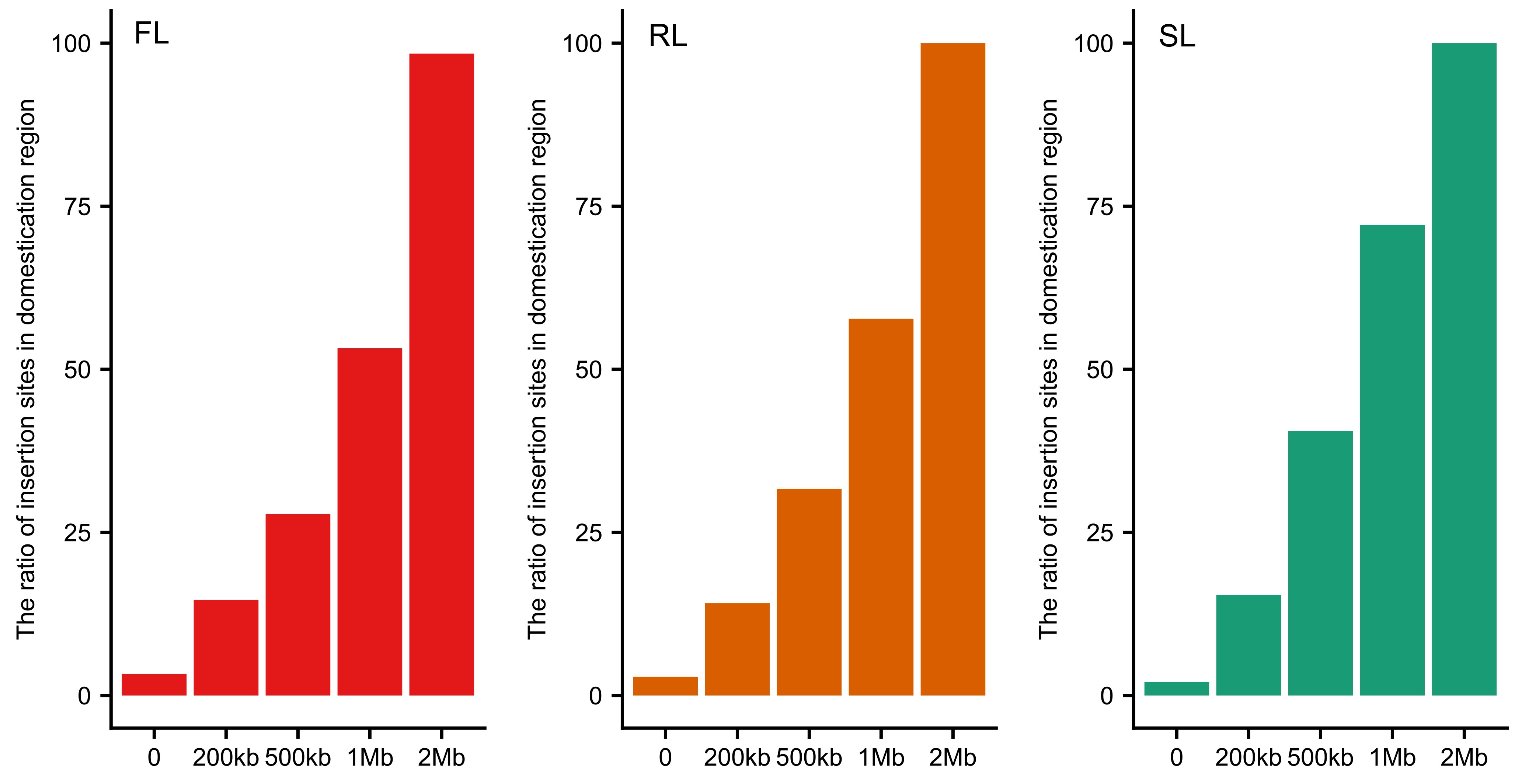

Supplement: Supplementary Figure 7 — The ratio of genomic positions of contigs in dispensable genomes located in or near domesticated regions. [file Image_7.jpeg]
